# Impact of Long-Term Swimming Exercise on Rat Femur Bone Quality

**DOI:** 10.3390/biomedicines12010035

**Published:** 2023-12-22

**Authors:** Laura Freitas, Andrea Bezerra, Ana Resende-Coelho, Maria Gomez-Lazaro, Leonardo Maciel, Tânia Amorim, Ricardo J. Fernandes, Hélder Fonseca

**Affiliations:** 1 Research Centre in Physical Activity, Health and Leisure (CIAFEL), Faculty of Sport, University of Porto, 4200-450 Porto, Portugal; dea.beatriz@hotmail.com (A.B.); ancatarina@gmail.com (A.R.-C.); yung_maciel@hotmail.com (L.M.); hfonseca@fade.up.pt (H.F.); 2 Laboratory for Integrative and Translational Research in Population Health (ITR), 4050-600 Porto, Portugal; 3 i3S—Institute for Research and Innovation in Health, University of Porto, 4200-135 Porto, Portugal; maria.glazaro@i3s.up.pt; 4 Postgraduate Nursing Program, Federal University of Sergipe, São Cristovão 49100-000, Brazil; 5 Department of Physiotherapy, Federal University of Sergipe, Lagarto 49400-000, Brazil; 6 Fame Laboratory, Department of Physical Education and Sport Science, University of Thessaly, 421-00 Trikala, Greece; tania_amorim@hotmail.com; 7 Centre of Research, Education, Innovation and Intervention in Sport (CIFI2D), Faculty of Sport, University of Porto, 4200-450 Porto, Portugal; ricfer@fade.up.pt; 8 Porto Biomechanics Laboratory (LABIOMEP), Faculty of Sport, University of Porto, 4050-313 Porto, Portugal

**Keywords:** bone mineral density, bone growth, bone microarchitecture, bone remodelling, non-weight bearing exercise

## Abstract

Considering the conflicting evidence regarding the potential long-term detrimental effect of swimming during growth on femur quality and fracture risk, our aim was to investigate the effect of eight months of swimming on femur quality. Twenty male eight-week-old *Wistar* rats were assigned into a swimming (SW; *n* = 10; 2 h/day, 5 days/week) or active control group (CG; *n* = 10, housed with running wheel) for eight months. Plasma osteocalcin and C-terminal telopeptide of type I collagen concentrations (ELISA) were assessed at baseline, four, and eight months of protocol. Femur structure (micro-computed tomography), biomechanical properties (three-point bending), and cellular density (histology) were determined after the protocol. SW displayed a lower uncoupling index, suggesting higher bone resorption, lower empty lacunae density, cortical and trabecular femur mass, femur length and cortical thickness, and higher cortical porosity than CG (*p* < 0.05). Although both biomarkers’ concentrations decreased in both groups throughout the experiment (*p* < 0.001), there were no significant differences between groups (*p* > 0.05). No differences were also found regarding biomechanical properties, bone marrow adiposity, and osteocyte and osteoclast densities (*p* > 0.05). Long-term swimming was associated with unbalanced bone turnover and compromised femur growth, lower femur mass, and deteriorated cortical bone microarchitecture. However, femur trabecular microarchitecture and biomechanical properties were not affected by swimming.

## 1. Introduction

Bone is an active and adaptable tissue that is influenced by external and internal mechanical stimulus from ground reaction forces and muscle contraction [1,2]. Exercise is an effective strategy to improve bone health by enhancing bone mass and structure, and is recommended by several guidelines [3,4] to counteract the negative effects of aging and bone health pathological conditions [5,6]. In particular, during childhood and adolescence, exercise promotes positive adaptations in bone mass and quality that can be maintained throughout most life [7,8]. Weight-bearing and high-impact exercises are considered as particularly osteogenic in comparison to non-weight bearing or low-impact exercises such as swimming [9,10]. Swimming is a non-weight bearing popular sport [11], developed mostly in a hypogravity environment [12], and it is contentious whether its practice has long-term negative effects on bone quality due to its non-osteogenic loading profile nature [6].

Swimmers have been shown to have lower bone mineral density (BMD) compared to both athletes engaged in weight-bearing exercise and non-athletes [13,14]. However, most evidence comes from observational cross-sectional studies with low-quality evidence [15], while data on the possible influence of swimming on bone geometry and microarchitecture, with both important variables of bone quality beyond BMD, are very limited [16]. There is also no long-term evidence from prospective studies following swimmers from childhood into adulthood determining if there is a causal relationship between swimming practice and low bone mass [17,18] or if the previously identified associations in cross-sectional studies are merely circumstantial. To address these literature gaps, several studies using animal models have been conducted with mixed findings [19]. Data have shown that swimming is associated with lower femur and vertebral trabecular microarchitecture [20], but other studies have reported improvements in femur trabecular microarchitecture [21] or have not observed any changes in tibia microarchitecture [22] and cortical geometry [23].

A possible confounder contributing to these contradictory findings is the use of swimming animal models with pathological conditions (e.g., osteoporosis and diabetes) [24,25] or sedentary animals confined to restrictive housing cage conditions as controls [21,26], opposed to their natural physically active behaviour in the wild [27]. In addition, swimming could elicit distinct bone adaptations according to the animal’s age and developmental phase [28]. Data on the long-term effect of swimming on bone are also very scarce since many studies are based on protocols only lasting a few weeks [19,29]. In fact, since no study has assessed the effect of swimming during the entire bone growth and development period, it was not possible to clarify the putative detrimental effects of swimming on bone health and to justify the implementation of adequate primary and secondary prevention strategies if these were confirmed. To address this need, the current study investigated the impact of eight months of swimming on rats’ femur bone mass, growth, microarchitecture, geometry, bone turnover markers, cellular density, and biomechanical properties, with the aim of testing the hypothesis that long-term swimming is detrimental to femur health.

## 2. Materials and Methods

### 2.1. Experimental Design

Based on an effect size of 1.18 [30] and considering α = 0.05 for a statistical power of 80% to detect a minimum relevant effect of swimming on femur volumetric bone mineral density (vBMD) between two groups, 20 male eight-week-old (323 ± 18.1 g) *Wistar Han* rats were obtained from Charles River laboratories (Les Oncins, France) and were individually housed in eurostandard type III H cages (floor area 800 cm^2^; Tecniplast, Buguggiate, Italy) in a temperature-controlled (24 ± 2 °C) 12 h inverted dark–light cycle vivarium. Ad libitum standard laboratory chow (Diete Standard 4RF21; Mucedola, S.r.l., Lombardia, Italy) and drinking water were provided to all animals. After two weeks of quarantine in a negative pressure ventilated cabinet, animals were randomly allocated through the “randombetween” function in Microsoft Excel into a swimmer group (SW; *n* = 10) or a physically active control group (CG; *n* = 10). The CG group was housed in cages equipped with a running wheel and revolution counter (Tecniplast, Buguggiate, Italy), mimicking voluntary physical activity in natural conditions. The SW group underwent a swimming exercise protocol over eight months. All animals were visually inspected daily for signs of stress, injury, or disease that could dictate withdrawal from the protocol accordingly to the humane endpoints defined a priori [31]. Food intake, running wheel activity, and body weight were also monitored throughout the experiment. At the beginning and at four months of protocol, 500 μL of blood from the tail vein was collected for quantification of biochemical markers of bone turnover. The local institutional Ethics Committee approved the study protocol (CEFADE 06 2021), according to the EU directive (2010/63/EU) and Portuguese law (DL 113/2013).

### 2.2. Swimming Exercise Protocol

**Figure 1:**
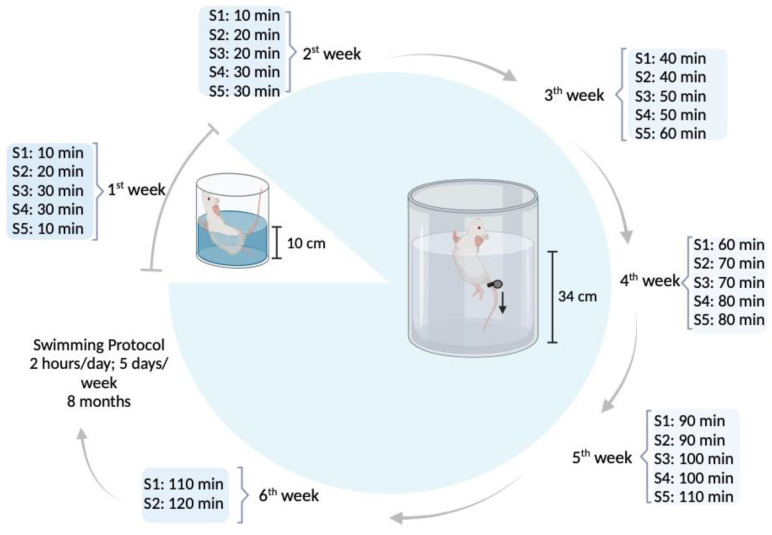
Water adaptation and experimental exercise protocol of the swimming group (image created in https://www.biorender.com/ (accessed on 15 November 2023).

Animals in the SW group underwent a swimming exercise protocol [32] for eight consecutive months. During the swimming sessions, each animal was placed inside a cylindrical plastic tube (20 cm diameter) in a warm water tank (107 cm diameter; 68 cm height; 30–32 °C). In the first week, a progressive water adaptation protocol was implemented [21,33], after which swimming duration increased progressively 10 min every two days until reaching 2 h/day of continuous swimming by the sixth week (Figure 1). Animals trained 5 days/week, for eight months with an overload of 3% body weight attached to their tail to prevent fluctuation [34,35]. Whenever animals were unable to keep themselves at the surface for >5 s, they were removed from water and allowed to rest for 1 min. One of the animals in the SW group was removed from the experiment due to its refusal to collaborate with the swimming protocol. After each swimming session, animals were dried and returned to their respective cages. The CG was maintained in cages with a running wheel throughout the eight months of the experiment.

### 2.3. Sacrifice and Sample Collection

All animals were sacrificed with an intraperitoneal overdose of ketamine (Nimatek, Dechra, Barcelona, Spain) and xylazine (Rompun, Bayer, Leverkusen, Germany). To eliminate the possible interference of the acute effects of exercise, three days before sacrifice, the swimming protocol was interrupted in the SW and the running wheels blocked in the CG [22]. During necropsy, blood from the abdominal vena cava (≈5 mL), heart, liver, gastrocnemius, and soleus muscles, as well as both femurs were collected and the tissues were weighed. Blood was collected into EDTA coated tubes (15452520, Fisher Scientific, Porto Salvo, Portugal), centrifuged at 2100× *g* for 10 min at 4 °C [36], and the plasma was separated and stored at −80 °C for quantification of biochemical markers of bone remodelling. The length of both femurs was determined from the greater trochanter to the lateral condyle with a digital calliper (resolution 0.01 mm; Powerfix, Diepenau, Germany) immediately after tissue harvest. The right femur was cleared of soft tissue and wrapped in saline-soaked gauze pads, sealed in plastic containers, and stored at −80 °C for micro-computed tomography (micro-CT) and biomechanical testing. The left femur was immediately fixed in 4% *w*/*v* buffered formaldehyde (PanReact AppliChem, 252931.1315) for histological analysis.

### 2.4. Femur Micro-Computed Tomography (Micro-CT)

Right femur bone mass (trabecular volumetric bone mineral density [Tb.vBMD] and cortical volumetric bone mineral density [Ct.vBMD]), microarchitecture (bone volume per tissue volume [BV/TV], trabecular thickness [Tb.Th], trabecular number [Tb.N], trabecular separation [Tb.Sp], connectivity density of trabeculae [Conn.D], cortical area per tissue area [Ct.Ar/Tt.Ar], cortical thickness [Ct.Th], and cortical porosity [Ct.Po]), and geometry (trabecular bone volume [Tb.V], cortical bone volume [Ct.V], marrow cross-sectional area [Ma.Ar], cortical cross-sectional area [Ct.Ar], and polar moment of inertia) were analysed by micro-CT. The right femur was scanned using the SkyScan 1276 micro-CT (Bruker, Kontich, Belgium), with the following parameters: voltage 85 kV; current 200 μA (with the aluminium filter, 1 mm); and 7.0 µm pixel size. The obtained images were reconstructed using the NRecon software Version 1.7.5.2. (SkyScan, Bruker, Kontich, Belgium). Reconstructed images were vertically oriented and saved in the transaxial plane using DataViewer Version 1.5.6.3. (SkyScan, Bruker, Kontich, Belgium). CTVox Version 3.3.0 r1412 (Bruker, Kontich, Belgium) was used to visualise the three-dimensional cross-sectional images. With CTAn Version 1.20.3 (SkyScan, Bruker, Kontich, Belgium), two- and three-dimensional models and the quantification of femur mass, microarchitecture, and geometry variables were obtained. Trabecular and cortical BMC were calculated by multiplying vBMD by their respective volumes.

Cortical bone parameters were analysed in the right femur diaphysis midshaft. A region of interest (ROI) of 900 slices from the growth plate (6 mm) was delimited and 100 slices were analysed (0.7 mm ROI). Trabecular bone variables were analysed in the left femur distal ephysis in a ROI starting 100 slices from the growth plate (0.7 mm), where 400 slices were investigated (2.8 mm ROI). All bone morphometric measurements and nomenclature are in accordance with the recommendations of the American Society for Bone and Mineral Research [37].

### 2.5. Femur Biomechanical Properties

Right femur diaphysis biomechanical properties were evaluated by a three-point bending test [38] using a servohydraulic testing machine (TIRATest 2705, TIRA, Schalkau, Germany). Bones were thawed at 4 °C overnight and maintained at room temperature wrapped in saline-soaked gauze prior to testing [39]. The distance between the equipment lower supports, where the femur was positioned, was determined with a digital calliper (resolution 0.01 mm; Powerfix, Diepenau, Germany) and a perpendicular bending load was applied halfway between this distance to the femur diaphysis in the antero-posterior direction (Figure S1, Supplementary Materials). A 0.1 mm/s preload was applied until the equipment reached 5 N, after which the velocity increased to 0.5 mm/s until bone fracture. Through the load–displacement curve (extrinsic bone properties), the maximum load (highest load reached during the test) was determined. The load–displacement curve was then converted to a stress–strain curve for determining femur diaphysis intrinsic properties, as described elsewhere [40] (Figure S1, Supplementary Materials). The following intrinsic bone properties were determined: (i) Young’s modulus (slope of the stress–strain curve in the elastic region; MPa); (ii) maximum stress (highest stress obtained; MPa); (iii) maximum strain (highest strain obtained; %); (iv) energy to yield (area under the stress–strain curve in the elastic region; MJ); (v) post-yield energy (area under the stress–strain curve from the Yield point until fracture; MJ); (vi) energy to fracture (sum of the energy to yield and post-yield energy; MJ); and (vii) brittleness coefficient (ratio between the strain at the Yield point and the strain at the bone fracture).

### 2.6. Biochemical Markers of Bone Turnover

Biochemical markers of bone remodelling were assessed in plasma samples at baseline, four, and eight months of the experimental protocol by quantification of osteocalcin (Rat-Mid Osteocalcin enzyme immunoassay, Immunodiagnostic Systems Ltd., Boldon, UK) and the C-terminal telopeptide of type I collagen (CTX; Rat-LapsR enzyme immunoassay, Immunodiagnostic Systems Ltd., Boldon, UK) by ELISA using a microplate reader (Labsystems iEMS, Labsystems Diagnostics, Helsinki, Finland) and following the manufacturers’ recommendations. All samples were thawed only once and were analysed in duplicate. Intra- and inter-assay coefficients of variation were 7.3% for osteocalcin and 3.4% and 10.0% for CTX. The uncoupling index was calculated at four and eight months of protocol according to Equation (S1), Supplementary Materials, as previously suggested [41,42]. Equation (S2), Supplementary Materials, was used to determine osteocalcin and CTX Z-scores for each animal after the exercise intervention [42].

### 2.7. Histomorphometry

The gastrocnemius and left femur were analysed by histology to determine muscle fibre cross-sectional area, osteocyte density, empty lacunae density, osteoclast activity, and bone marrow adiposity. Following appropriate fixation, the left femur was decalcified in 10% *w*/*v* ethylenediaminetetraacetic acid (EDTA; D/0450/50, Fisher Scientific, Porto Salvo, Portugal) in phosphate-buffered saline (PBS; BP2944100, Fisher Scientific, Porto Salvo, Portugal). This was completed for approximately 30 days, under constant agitation in a platform rocker (STR6, Stuart Scientific, UK), and complete decalcification was confirmed through the ammonium oxalate test [43]. Subsequently, bones underwent histological processing by dehydration in graded ethanol solutions (E/0650DF/C17, Fisher Chemical, Waltham, MA, USA), clearing in xylene (6615, Thermo Scientific, Waltham, MA, USA) and embedding in paraffin wax (8336, Thermo Scientific, Waltham, MA, USA). The gastrocnemius was separated in white and red regions, and processed as described above without the initial decalcification step. Five µm thickness sections were obtained with a rotary microtome (Leica RM2125 RT, Leica Microsystems; Nussloch, Germany). Slides of the femur diaphysis, distal epiphysis, and gastrocnemius mid-portion were stained with hematoxylin (72704, Thermo Scientific, Waltham, MA, USA) and eosin (71204, Thermo Scientific, Waltham, MA, USA) after adequate deparaffinisation and rehydration.

Osteoclasts were identified by tartrate-resistant acid phosphatase (TRAP) staining using a commercial Kit (387A, Sigma-Aldrich, Taufkirchen, Germany) according to the manufacturer’s instructions [44]. All slides from the femur and gastrocnemius were examined under a light microscope and images captured with a coupled digital camera (Axio Imager A1, Carl Zeiss, Göttingen, Germany). Images were analysed through ImageJ (version 1.53K, National Institutes of Health, Bethesda, MD, USA) for: (i) muscle fibre cross-sectional area (µm^2^), determined as the average of 350 fibres from each animal taken from 35 representative images distributed through the muscle; (ii) osteocyte density (N.Ot/Ct.Ar; N/mm^2^), determined as the number of osteocytes identified per cortical bone area in femur diaphysis transverse sections (eight images were analysed per animal); (iii) empty lacunae density, determined as the number of empty lacunae per cortical bone area (N.Lc/Ct.Ar; N/mm^2^); (iv) osteoclast density (N.Oc/T.Ar; N/mm^2^), determined as the number of osteoclasts per tissue area in the femur distal epiphysis growth plate region (≈14 images per animal); and (v) bone marrow adiposity (Ad.Ar/Ma.Ar; %), determined as the fraction of adipocytes covered area (Ad.Ar; μm^2^) relative to bone marrow area (Ma.Ar; μm^2^) assessed in the femur distal epiphysis (four images per animal). The intra-observer coefficients of variation were ~5% for N.Ot/Ct.Ar, 10% for N.Lc/Ct.Ar, 3% for Ad.Ar, and ~1% for N.Oc/T.Ar.

### 2.8. Statistical Analysis

Statistical analyses were performed using SPSS Statistical software (version 29.0), with a significance level of *p* < 0.05. Data were reported as mean ± standard deviation (SD). Outliers were detected and if they significantly influenced the results, they were excluded from the analysis, such as in the instance of Conn.D and Ct.Po. Four animals (two per group) were excluded from the biomechanical testing analysis due to the atypical stress–strain curves obtained. Variables’ normality was verified by the Shapiro–Wilk test and in the case of a non-normal distribution, an appropriate transformation was applied. If the transformation was ineffective, testing was conducted with bootstrapping (1000 samples and 95% confidence interval), as in the cases of tibia midshaft Ct.vBMD and N.Oc/T.Ar. The effect of swimming on bone biomarkers was determined through linear mixed models. Independent *t*-tests were performed to compare all variables between the CG and SW groups and the effect size was reported as Cohen’s d when *p* < 0.05 [45].

## 3. Results

### 3.1. Food Intake, Physical Activity, and Morphometry

The food intake, physical activity, and morphometry of the CG and SW groups are displayed in Table 1. The initial body weight was similar between groups, whereas, at the end of the protocol, a lower body weight and body weight variation were found in SW compared to CG, despite the amount of weekly food intake being similar. There were no differences in heart and liver mass, as well as in the gastrocnemius white portion muscle fibre cross-sectional area between groups. Pooled gastrocnemius and soleus mass, and gastrocnemius red portion fibre cross-sectional area were lower for SW in comparison to the controls. Animals from the SW group also tended to display a higher number of gastrocnemius fibres with a smaller size (Figure S2, Supplementary Materials). During the experimental protocol, an average of ~9 km/week on the activity wheels was observed in animals from the CG.

### 3.2. Femur Growth Mass, Geometry, Microarchitecture, and Biomechanical Properties

**Figure 2:**
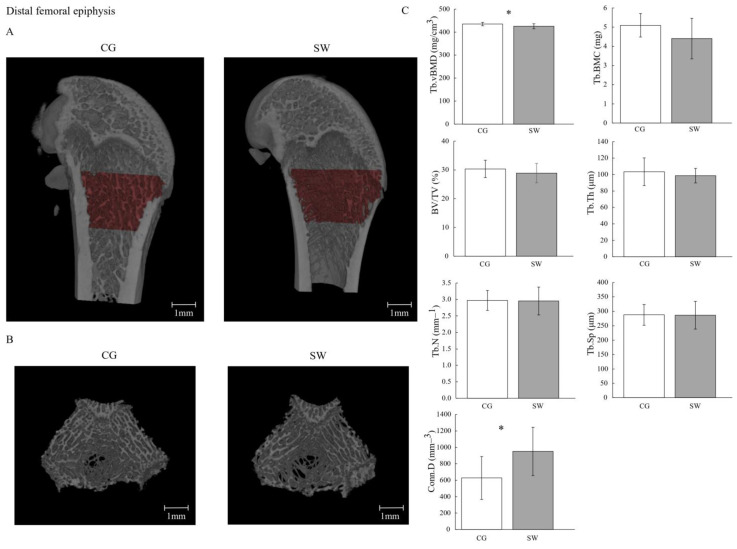
Trabecular mass and microarchitecture at distal femoral epiphysis in growing rats between the control (*n* = 10, except for trabeculae connectivity density *n* = 9 after removing the outliers) and swimming groups (*n* = 9, except for trabeculae connectivity density *n* = 7 after removing the outliers) after eight months of the experiment (**C**), with the representative micro-computed tomography images of the sagittal (**A**) and transverse planes (**B**). Data are displayed as mean ± SD. Legend: Active control group (CG); bone volume fraction (BV/TV); trabeculae connectivity density (Conn.D); trabecular bone mineral content (Tb.BMC); trabecular number (Tb.N); trabecular thickness (Tb.Th); trabecular separation (Tb.Sp); trabecular volumetric bone density (Tb.vBMD); * *p* < 0.05.

**Figure 3:**
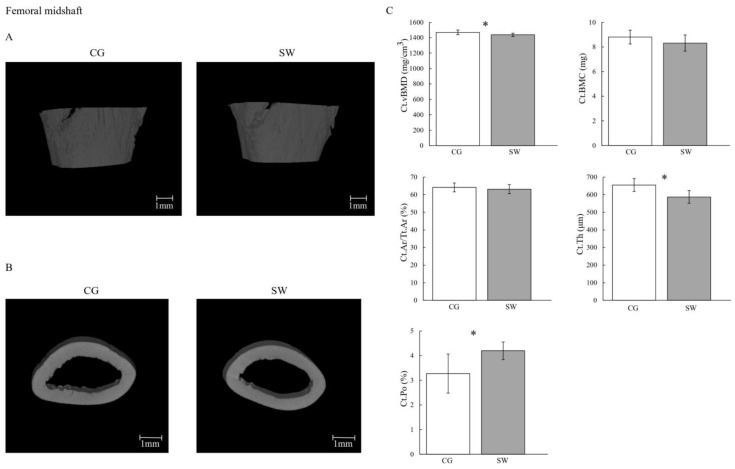
Cortical mass and microarchitecture at the femoral midshaft in growing rats between the control (*n* = 10, except for cortical porosity *n* = 9 after removing the outliers) and swimming (*n* = 9, except for cortical porosity *n* = 8 after removing the outliers) groups after eight months of the experiment (**C**), with the representative micro-computed tomography images of the sagittal (**A**) and transverse planes (**B**). Data are displayed as mean ± SD. Legend: Active control group (CG); cortical area fraction (Ct.Ar/Tt.Ar); cortical bone mineral content (Ct.BMC); cortical porosity (Ct.Po); cortical thickness cortical (Ct.Th); volumetric bone density (Ct.vBMD); swimming group (SW); * *p* < 0.05.

Figure 2 and Figure 3 display bone mass and microarchitecture variables, as well as the representative 3D images of distal femur epiphysis and midshaft (trabecular and cortical bone, respectively).

Right and left femur lengths were lower in SW compared to CG (Table 2). The Tb.vBMD (CG: 435.26 ± 7.32 vs. SW: 425.60 ± 11.24 mg/cm^3^; *p* = 0.036, d = 1.06) and Ct.vBMD (CG: 1469.20 ± 30.30 vs. SW: 1437.70 ± 21.60 mg/cm^3^; *p* = 0.019, d = 1.19) were lower in SW compared to the CG group. No differences were observed at Tb.BMC (CG: 5.10 ± 0.61 vs. SW: 4.41 ± 1.06 mg; *p* = 0.096) and Ct.BMC (CG: 8.80 ± 0.56 vs. SW: 8.32 ± 0.66 mg; *p* = 0.100). Despite higher Conn.D in SW (CG: 627.26 ± 261.63 vs. SW: 950.96 ± 294.83 mm^−3^; *p* = 0.036, d = −1.17), no differences were found between groups regarding femur distal epiphysis BV/TV (CG: 30.39 ± 3.02 vs. SW: 28.91 ± 3.36%; *p* = 0.325), Tb.Th (CG: 98.60 ± 8.75 vs. SW: 103.36 ± 16.93 μm; *p* = 0.488), Tb.N (CG: 2.971 ± 0.303 vs. SW: 2.952 ± 0.423 mm^−1^; *p* = 0.912), Tb.Sp (CG: 287.60 ± 35.95 vs. SW: 286.53 ± 47.98 μm; *p* = 0.812), or Ct.Ar/Tt.Ar (CG: 64.17 ± 2.50 vs. SW: 63.11 ± 2.56%; *p* = 0.374). In the femur midshaft, Ct.Th was lower in the SW group (CG: 654.95 ± 36.68 vs. SW: 587.18 ± 36.25 μm; *p* = 0.001, d = 1.86), while Ct.Po was higher (CG: 3.270 ± 0.790 vs. SW: 4.197 ± 0.359%; *p* = 0.008, d = −1.48). For any of the remaining geometric (Tb.V, Ct.V, Ma.Ar, Ct.Ar, and polar moment of inertia) and biomechanical properties (maximum load, Young’s modulus, maximum stress, maximum strain, energy to Yield point, post-Yield point energy, energy to fracture, and brittleness coefficient) variables assessed, similar values were identified between groups.

### 3.3. Biochemical Markers of Bone Turnover

**Figure 4:**
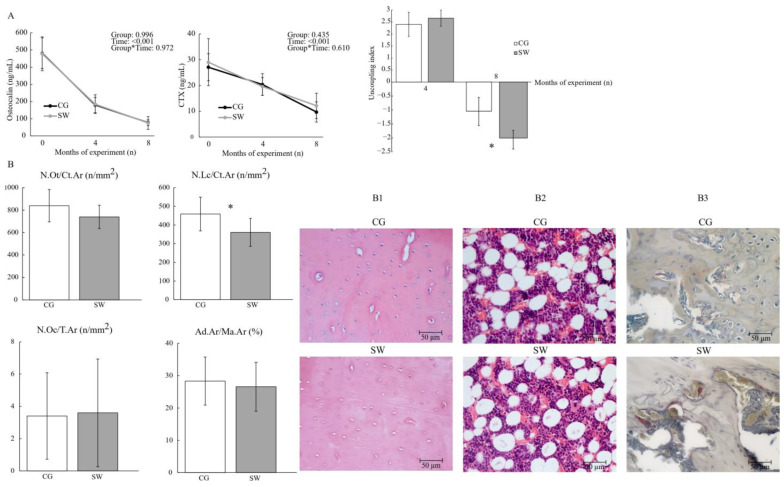
Bone turnover assessed by the biochemical markers and bone turnover uncoupling index (**A**) and femur osteocyte, lacunae, and osteoclast densities (**B**) between the control (*n* = 10) and swimming (*n* = 9) groups after eight months of the experiment, with representative images of haematoxylin and eosin staining for osteocyte, lacunae densities (**B1**), and bone marrow adiposity (**B2**), and tartrate-resistant acid phosphatase staining for osteoclast density (**B3**). Data are displayed as mean ± SD. Legend: Active control group (CG); bone marrow adiposity (Ad.Ar/Ma.Ar); C-terminal telopeptide of type I collagen (CTX); empty lacunae density (N.Lc/Ct.Ar); osteocyte density (N.Ot/Ct.Ar); osteoclast density (N.Oc/T.Ar); swimming group (SW); * *p* < 0.05.

Both groups displayed similar plasma osteocalcin concentrations at baseline (CG: 480.75 ± 89.17 vs. SW: 477.77 ± 98.24), four months (CG: 179.84 ± 44.39 vs. SW:184.64 ± 54.49), and eight months (CG: 77.15 ± 16.76 vs. SW:75.00 ± 36.95 ng/mL) of the protocol (Figure 4). There were also no differences in CTX concentrations in any of the experimental moments (baseline CG: 27.10 ± 5.27 vs. SW: 29.057 ± 9.108 ng/mL; four months CG: 20.37 ± 4.20 vs. SW: 19.62 ± 3.45 ng/mL; eight months CG: 9.71 ± 3.90 vs. SW: 12.13 ± 4.90 ng/mL; *p* = 0.435). Even if both bone biomarkers expressed no effect of experiment time and group, their concentration decreased in both groups throughout the experiment. A positive uncoupling index was observed at four months of the experiment, but without differences between groups (CG: 2.40 ± 0.51 vs. SW: 2.66 ± 0.33; *p* = 0.257). However, the uncoupling index at the end of the experiment was lower in the SW group compared to CG, suggesting a higher imbalance towards bone resorption in this group (CG: −1.05 ± 0.71 vs. SW: −2.13 ± 0.70; *p* = 0.001, *d* = 1.53).

### 3.4. Femur Histological Analysis

Osteocyte density (CG: 838.81 ± 144.48 vs. SW: 740.23 ± 104.27 n/mm^2^; *p* = 0.111), bone marrow adiposity at the femur diaphysis (CG: 28.29 ± 7.42 vs. SW: 26.56 ± 7.55%; *p* = 0.621), and osteoclast density at the femur distal epiphysis (CG: 3.41 ± 2.68 vs. SW: 3.60 ± 3.34 n/mm^2^; *p* = 0.897) were similar between groups (Figure 4). Empty lacunae density was lower in the SW group compared to CG (CG: 458.82 ± 90.510 vs. SW: 361.03 ± 74.71 n/mm^2^; *p* = 0.021, *d =* 1.08).

## 4. Discussion

Considering the lack of longitudinal data on the effects of swimming throughout most of the bone growth period, the aim of the current study was to investigate if long-term swimming had detrimental effects on femur bone quality in rat models. Our results showed that eight months of swimming had a deleterious effect on femur mass, growth, cortical microarchitecture, and bone turnover. However, no changes were found in femur geometry, biomechanical properties, osteocyte density, osteoclast density, or bone marrow adiposity.

Over the eight-month experiment, the concentration of biomarkers of bone formation and resorption decreased in both groups (CG and SW), which was expected since it reflects the slowing in the animal growth rate. Additionally, swimming was not associated with significantly altered variations in osteocalcin or CTX concentrations compared to physically active controls. The number of osteoclasts identified in the femur distal epiphysis, an indicator of local bone resorption, was also unaffected by swimming. Nonetheless, the bone turnover uncoupling index was lower in the SW group, suggesting an imbalance towards higher bone resorption. Although bone remodelling is crucial for adequate maintenance of blood calcium concentration and to ensure bone tissue renewal and maintenance of biomechanical properties, chronic states of unbalanced bone turnover lead to net bone loss and impaired bone quality [46]. Our results indicate that there were no differences in osteocalcin and CTX concentration between groups, whereas bone turnover tended to be chronically unbalanced in swimming animals, favouring bone loss. The evidence on the effect of swimming on bone turnover markers is conflicting [22,25] and could be related to several factors, like the use of animal models in different developmental stages, with normal or pathological conditions, swimming protocol characteristics, or the intrinsic lability of biochemical markers of bone turnover potentially affected by methodological variables such as time of day and diet. For instance, young male rats swimming for eight weeks displayed no differences in biochemical markers of bone formation and resorption [22], while after eight weeks of swimming, female rats showed higher serum osteocalcin and lower CTX concentrations, indicating increased bone formation [25].

The effect of swimming on bone turnover resulted in lower femur mass, particularly lower trabecular and cortical vBMD, and compromised femur growth, reflected by the lower femur length, as well as changes in cortical microarchitecture, specifically lower Ct.Th and a higher Ct.Po. The previous literature shows contradictory results on femur bone mass and cortical microarchitecture following swimming. In two previous studies with a duration of eight weeks of swimming, one observed no effect of swimming on femur cortical vBMD [47], while the other identified a reduction in cortical areal BMD [22]. In addition, 16 weeks of swimming resulted in reduced femur trabecular vBMD and Ct.Th [48], whereas after 10 weeks of swimming, female rats displayed no changes in Ct.Th [49]. Several studies with swimming protocols of different durations also reported no impact of swimming on femur size [21,48,50,51], whereas one study with a particularly high training volume (6 h/day) reported that swimming impaired femur length, in agreement with our findings [52].

Animals from the SW group also exhibited lower body weight and femur length compared to CG, despite similar food intake between the groups. These results suggest a possible effect of swimming on inadequate energy availability due to the high energy expenditure resulting from the daily swimming exercise protocol, which could have hindered the animals’ growth. There is broad evidence in the literature that low energy availability is associated with compromised bone growth, unbalanced bone remodelling, lower bone mass, and deteriorated geometry and microarchitecture in rodents [53,54]. Nevertheless, bone marrow adiposity, which has also been increased in conditions associated with energy deficiency [55], remained unaffected by swimming. This result suggests that the effect of swimming on femur growth restriction might have occurred in an earlier phase of their development since, at the sacrifice moment, there were no signs of either altered bone marrow adiposity or biochemical markers of bone formation.

Cellular density is also a key feature determining bone quality due to the role of osteocytes on local bone remodelling, mechanotransduction, and bone tissue hydration [1]. Moreover, osteocyte viability is negatively affected by mechanical unloading [56] and, remarkably, osteocyte density was unaffected after eight months of swimming. Again, the lack of differences in osteocyte viability between SW and CG proposes that the mechanisms favouring the low bone mass and cortical bone deterioration identified in swimmers might have occurred mostly during the earlier phases of the animals’ development and were not noticeable at the time of sacrifice. Notwithstanding the negative adaptations observed in the femur cortical bone, no changes were observed in most of the trabecular microarchitecture variables assessed (BV/TV, Tb.N, Tb.Th, and Tb.Sp), while, interestingly, trabecular connectivity was found to be higher in SW. It is possible that the higher trabecular connectivity identified in SW could be attributable to their lower femur size [57].

Despite evidence of some detrimental effects of swimming on femur mass, growth, and cortical microarchitecture, the current study results did not reveal any negative effect on the biomechanical properties of the femur diaphysis assessed by three-point-bending test. This finding advocates that, even though swimming tends to induce changes in femur size and structure, it is likely that some compensatory adaptations also occur, preventing significant decreases in femur resistance to fracture. Young male rats submitted to eight weeks of swimming showed no differences in femur geometry and on most of the biomechanical variables assessed, with only increases in Young’s modulus being detected [51]. Another study with a similar swimming duration observed that swimming was associated with increases in post-yield energy absorption [22]. Interestingly, animals from the SW group had a lower pooled gastrocnemius and soleus muscle mass, as well as a lower muscle fibre cross-sectional area in the gastrocnemius, proposing that mechanical loading derived from muscle tension could also have been lower in SW compared to the CG. Therefore, the negative effects of swimming detected on the femur could be associated with both the lower gravitational forces resulting from the aquatic environment and muscle contractions of lower intensity [12].

This current study has several limitations that need to be taken into consideration in the analysis of the results. Considering that swimming is a forced exercise protocol performed in an unhabitual environment for the animal, it is possible that stress associated with this exercise protocol, despite the gradual habituation, could also have contributed to a negative impact on bone health. The absence of measurements of stress surrogates hinders a thorough appreciation of this possible influence. Nevertheless, this study also has several virtues, such as including the longest swimming protocol in the literature, exposing animals to daily swimming sessions over eight months, mimicking the exposure to swimming from childhood until adulthood. This study also analysed the effect of swimming not only on bone mass but on several properties determining bone quality, enabling a thorough perspective regarding the effects of swimming on femur health.

## 5. Conclusions

Long-term swimming during most of the growth and development period in male rats is associated with unbalanced bone turnover, reduced femur growth, lower bone mass, and impaired cortical bone microarchitecture. Nevertheless, swimming does not seem to negatively affect distal femur trabecular microarchitecture or femur diaphysis biomechanical properties.

## Figures and Tables

**Table 1:** Morphometry, food intake, and physical activity recorded during the eight months of the experiment.

Variables (Units)	Experimental Groups		Independent *t*-Test
	CG (*n* = 10)	SW (*n* = 9)	*p* Value (Cohen’s d)
Initial body weight (g)	315.5 ± 12.4	332.2 ± 23.0	0.079
Final body weight (g)	533.70 ± 48.15	477.33 ± 47.73	0.020 (1.18)
Body weight variation (%)	69.07 ± 12.29	43.63 ± 9.57	<0.001 (2.31)
Food intake (kcal/week)	666.13 ± 73.73	636.58 ± 36.27	0.292
Physical activity (km/week)	9.09 ± 9.36	--	--
Heart mass (g)	1.24 ± 0.11	1.30 ± 0.08	0.205
Liver mass (g)	15.31 ± 1.44	14.46 ± 2.00	0.302
Muscle mass ^1^ (g)	6.54 ± 0.47	5.98 ± 0.32	0.010 (1.14)
CSA gastrocnemius red portion (µm^2^)	2752 ± 206	2529 ± 253	0.049 (0.972)
CSA gastrocnemius white portion (µm^2^)	3482 ± 424	3172 ± 443	0.137

Data are displayed as mean ± SD. Legend: Cross-sectional area (CSA); control group (CG); pooled gastrocnemius and soleus muscle mass (Muscle mass ^1^); swimming group (SW).

**Table 2:** Femur growth, geometry, and biomechanical properties in the control and swimming groups at the end of the eight months of the exercise protocol.

Femur Health	Variables (Units)	Experimental Groups		Independent *t*-Test
		CG	SW	*p* Value (Cohen’s d)
Growth	Right femur length (mm)	40.80 ± 1.07	39.75 ± 1.23	0.045 (0.91)
	Left femur length (mm)	40.76 ± 0.89	39.71 ± 1.24	0.047 (0.99)
Geometry	Tb.V (mm^3^)	11.70 ± 1.28	10.33 ± 2.35	0.128
	Ct.V (mm^3^)	5.99 ± 0.29	5.79 ± 0.47	0.276
	Ct.Ar (mm^2^)	8.43 ± 0.41	8.15 ± 0.67	0.278
	Ma.Ar (mm^2^)	29.96 ± 9.97	36.23 ± 7.42	0.142
	Polar moment of inertia (mm^5^)	18.20 ± 1.15	17.47 ± 2.65	0.509
Biomechanical properties	Maximum load (N)	184 ± 47	191 ± 33	0.703
	Young’s modulus (Mpa)	10133 ± 1378	10132 ± 1046	0.998
	Maximum stress (Mpa)	196 ± 47	201 ± 28	0.780
	Maximum strain (%)	2.70 ± 0.05	3.00 ± 0.06	0.298
	Energy to Yield point (MJ)	1.70 ± 0.31	1.64 ± 0.20	0.684
	Post-Yield point energy (MJ)	1.85 ± 1.16	2.11 ± 0.71	0.622
	Energy to fracture (MJ)	3.00 ± 1.36	3.24 ± 1.17	0.689
	Brittleness coefficient	0.72 ± 0.15	0.66 ± 0.09	0.318

Data are displayed as mean ± SD. All variables with *n* = 10 for CG (except for energy to Yield point, post-Yield point energy, and brittleness coefficient, *n* = 8) and *n* = 9 for SW (except for energy to Yield point, post-Yield point energy, and brittleness coefficient, *n* = 7). Legend: Control group (CG); cortical mean total cross-sectional area (Ct.Ar); cortical bone volume (Ct.V); bone marrow mean total cross-sectional area (Ma.Ar); swimming group (SW); trabecular bone volume (Tb.V).

## Data Availability

All data relevant to this study are included in this article or in Supplementary Materials.

## Supplementary Materials

The following supporting information can be downloaded at https://www.mdpi.com/article/10.3390/biomedicines12010035/s1: Figure S1: The preparation of the right femur in the antero-posterior position for the three-point bending test and the assessment of the load–displacement and stress–strain curves with respective equations; Equations (S1) and (S2): Calculation of the uncoupling index and Z-score, respectively; Figure S2: The absolute frequencies regarding the cross-sectional area of gastrocnemius red and white portions in male rats comparing the active control group with the swimming exercise group for eight months.
