# Supplementary material for: Impact of Long-Term Swimming Exercise on Rat Femur Bone Quality
Source: Biomedicines. 2023 Dec 22;12(1):35. doi: 10.3390/biomedicines12010035 (PMC10813774; doi:10.3390/biomedicines12010035)
Supplement: Supplementary file 1 [file biomedicines-12-00035-s001.zip › biomedicines-2773057-supplementary.pdf]

Impact of long-Term Swimming Exercise on Rat Femur Bone Quality

Biomedicines

Laura Freitas [1,2,*], Andrea Bezerra [1,2], Ana Resende-Coelho [1,2], Maria Gomez-Lazaro [3], Leonardo Maciel [1,2,4,5], Tânia Amorim [6], Ricardo J. Fernandes [7,8] and Hélder Fonseca [1,2]

1 Research Centre in Physical Activity, Health and Leisure (CIAFEL), Faculty of Sport, University of Porto, 4200-450 Porto, Portugal; dea.beatriz@hotmail.com (A.B.); ancatarina@gmail.com (A.R.-C.); yung_maciel@hotmail.com (L.M.); hfonseca@fade.up.pt (H.F.)
2 Laboratory for Integrative and Translational Research in Population Health (ITR), 4050-600 Porto, Portugal
3 i3S—Institute for Research and Innovation in Health, University of Porto, 4200-135 Porto, Portugal; maria.glazaro@i3s.up.pt
4 Postgraduate Nursing Program, Federal University of Sergipe, São Cristovão 49100-000, Brazil
5 Department of Physiotherapy, Federal University of Sergipe, Lagarto 49400-000, Brazil
6 Fame Laboratory, Department of Physical Education and Sport Science, University of Thessaly, 421-00 Trikala, Greece; tania_amorim@hotmail.com
7 Centre of Research, Education, Innovation and Intervention in Sport (CIFI2D), Faculty of Sport, University of Porto, 4200-450 Porto, Portugal; ricfer@fade.up.pt
8 Porto Biomechanics Laboratory (LABIOMEP), Faculty of Sport, University of Porto, 4050-313 Porto, Portugal
* Correspondence: laura_c_freitas@hotmail.com

Supplementary Materials

Figure S1: Preparation of the right femur in antero-posterior position for the three-point bending test and the assessment of the load-displacement and stress-strain curves with respective equations. 1: The application of the three-point bending test to obtain load-displacement curve. 2: The calculation of moment of inertia through femoral midshaft dimensions from micro-computed tomography image to assess stress-strain curve. 3: The stress-strain curve measured by stress and strain respective equations. The illustration was developed using BioRender software. Lateromedial femur diameter (A); craniocaudal bone diameter (B); distance from the load application to the bone neutral axis (B/2); Lateromedial marrow diameter (C); craniocaudal marrow diameter (D); load applied (F); moment of inertia (I); distance between the supports (L); displacement (Z).......... 3

Equations S1 and S2 related to the calculation of uncoupling index and Z-score 4

Figure S2: Fibre cross-sectional area of gastrocnemius red and white portions in growing rats between control (n= 10) and swimming (n= 9) groups after eight months of experiment (upper panel), with representative images of hematoxylin and eosin staining of transversal sections of previous muscles (lower panel). Data are displayed in n (absolute frequencies). Cross-sectional area (CSA); control group (CG); swimming group (SW). 5

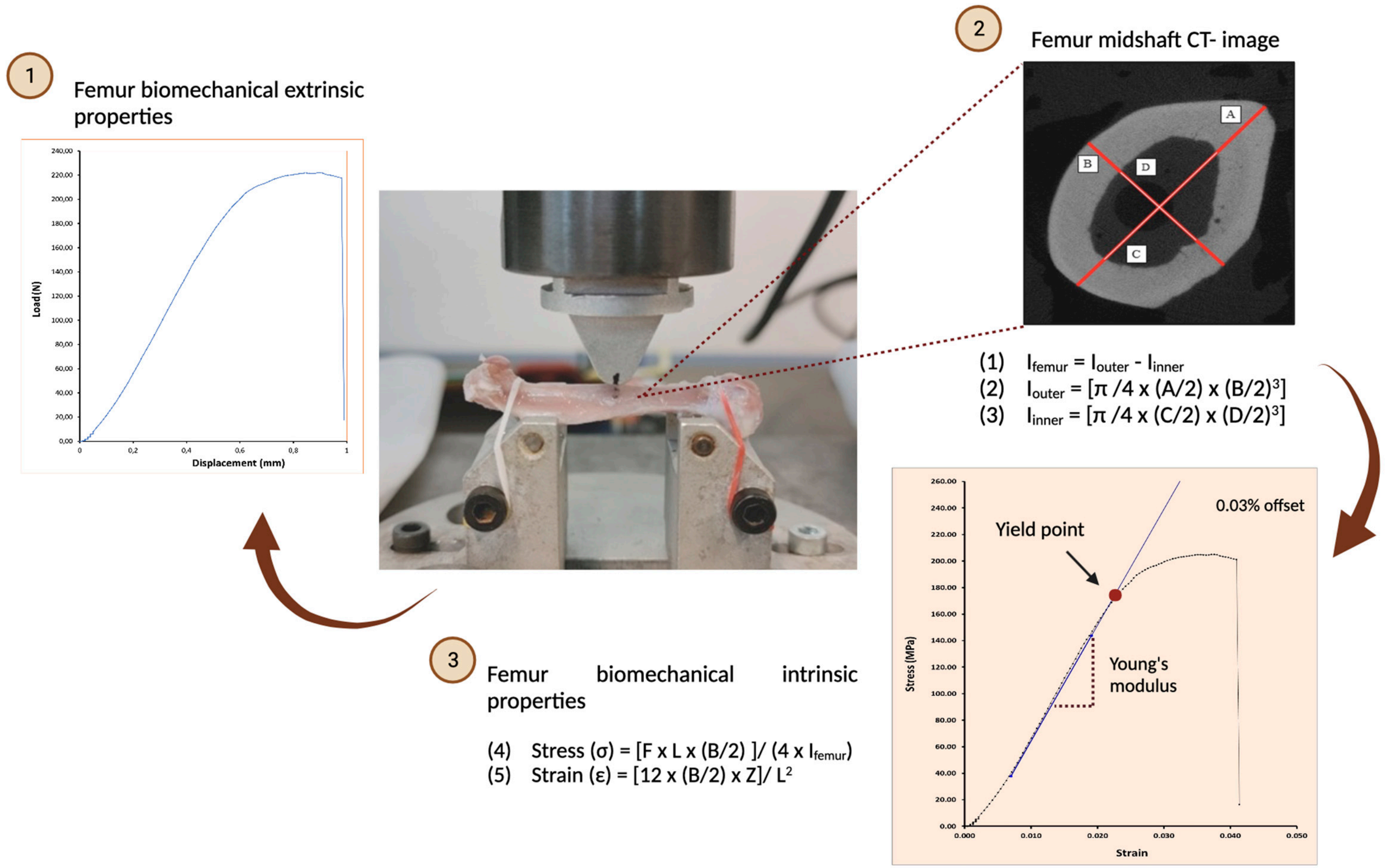

Figure S1: Preparation of the right femur in antero-posterior position for the three-point bending test and the assessment of the load-displacement and stress-strain curves with respective equations. 1: The application of the three-point bending test to obtain load-displacement curve. 2: The calculation of moment of inertia through femoral midshaft dimensions from micro-computed tomography image to assess stress-strain curve. 3: The stress-strain curve measured by stress and strain respective equations. The illustration was developed using BioRender software. Lateromedial femur diameter (A); craniocaudal bone diameter (B); distance from the load application to the bone neutral axis (B/2); Lateromedial marrow diameter (C); craniocaudal marrow diameter (D); load applied (F); moment of inertia (I); distance between the supports (L); displacement (Z).

Equations S1 and S2 related to the calculation of uncoupling index and Z-score

Equation (S1):

$$\text{Uncoupling index} = \text{Z-score}_{\text{Osteocalcin}} / \text{Z-score}_{\text{CTX}}$$

Equation (S2):

$$\text{Z-score} = (\text{Animal value} - \text{Mean}_{\text{baseline}}) / \text{Standard Deviation}_{\text{baseline}}$$

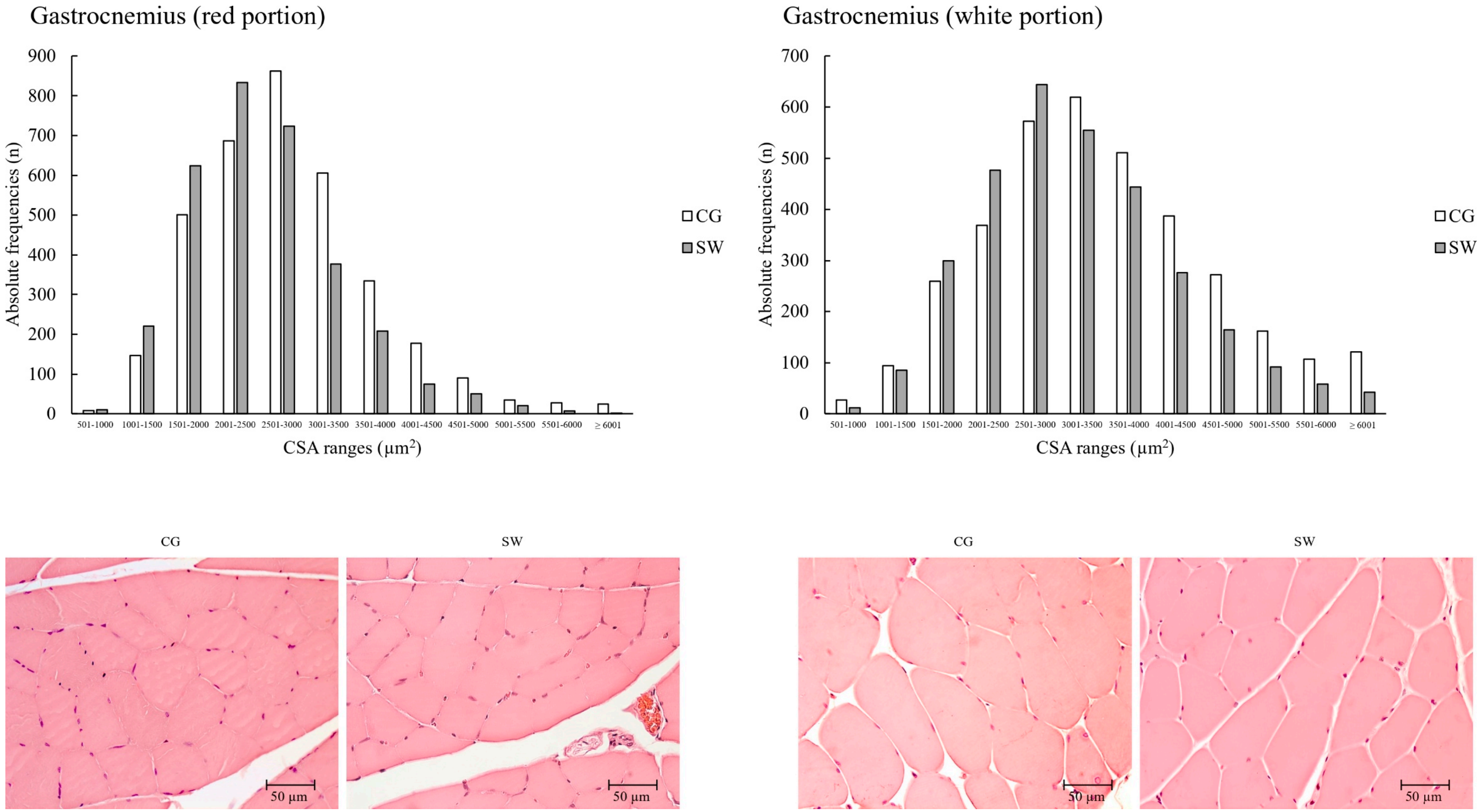

Figure S2: Fibre cross-sectional area of gastrocnemius red and white portions in growing rats between control (n= 10) and swimming (n= 9) groups after eight months of experiment (upper panel), with representative images of hematoxylin and eosin staining of transversal sections of previous muscles (lower panel). Data are displayed in n (absolute frequencies). Cross-sectional area (CSA); control group (CG); swimming group (SW).
